# Editor's Letter-Box

**Published:** 1895-05-04

**Authors:** 


### EDITOR'S LETTER-BOX.

[Our correspondents are reminded that prolixity is a great bar to publication, and that brevity of style and conciseness of statement greatly facilitate early insertion.]

Mr. G. E. Adams, secretary of the West Ham Hospital, Stratford, E., writes: In your report of the opening of the new wing of this hospital in Saturday's issue of The Hospital you state Mr. Passmore Edwards gave £2,000. This is incorrect. Mr. Edwards has given £3,000, and at the opening ceremony promised to defray the cost of furnishing the "Eleanor Edwards" Ward. Mr. J. R. Roberts has also kindly promised to bear the expense of furnishing the "J. R. Roberts" Ward, in addition to his handsome donation of £1,000. The wing is named "Passmore Edwards Wing," and the total cost is £3,750. Will you kindly make the necessary correction in your next issue, and oblige.

